# Association between neurological outcome and poststroke comorbid mood and anxiety disorders: A real‐life experience

**DOI:** 10.1002/brb3.2158

**Published:** 2021-05-05

**Authors:** Yolaine Rabat, Richard Houeze, Sharmila Sagnier, Stephane Olindo, Mathilde Poli, Sabrina Debruxelles, Pauline Renou, François Rouanet, Sylvie Berthoz, Igor Sibon

**Affiliations:** ^1^ Univ. Bordeaux CNRS EPHE INCIA UMR 5287 Bordeaux France; ^2^ CHU Bordeaux Stroke Unit Department of Neurology Bordeaux France; ^3^ Centre Hospitalier de la Police Républicaine Cotonou Bénin; ^4^ Department of Psychiatry for Adolescents and Young Adults Institut Mutualiste Montsouris Paris France

**Keywords:** anxiety, cognition, depression, functional outcome, pain, sleep, stroke

## Abstract

**Introduction:**

Poststroke depression (PSD) and anxiety (PSA) are prevalent and have a strong impact on functional outcome. Beside stroke severity, little is known on their clinical determinants. This study investigated the association between stroke mechanism, neurological poststroke complications and remaining vascular risk factors and the presence of comorbid PSD and PSA, termed poststroke emotional distress (PSED).

**Methods:**

This was a retrospective analysis of a prospectively compiled medical records database of consecutive patients evaluated during a follow‐up visit 3‐ to 4‐month poststroke. HAD scale was used to define PSED category (PSD+PSA vs. NoPSD+NoPSA). Stroke mechanism and poststroke complications were identified clinically or using appropriate scales. Their association with PSED was tested using a multivariate logistic regression model.

**Results:**

The sample included 2,300 patients (male: 64.8%); 19% had a PSED and 56.39% were free of any depression or anxiety. The most frequent poststroke complications were fatigue/fatigability (58.4%), sleep problems (26.7%), and pain (20.4%). While no association was observed between PSED and stroke mechanism, higher functional disability (OR:1.572), lower cognitive abilities (OR:0.953), sleep problems (OR:2.334), pain (OR:1.478), fatigue/fatigability (OR:2.331), and abnormal movements (OR:2.380) were all independent risk factors. Persisting tobacco consumption (OR:1.360) was the only vascular significant risk factor.

**Conclusions:**

The frequency of comorbid PSED remains high (1/5 patient) despite improved awareness of these conditions. The association between poststroke complications and the presence of PSED emphasizes the need for standardized neurological and psychological evaluations at follow‐up. These results foster the need to improve the management of addictive behaviors to reduce the burden of PSED.

## Graphical abstract

The frequency of comorbid PSED remains high (1/5 patient) despite improved awareness of these conditions. Most of poststroke complications were independently associated with the presence of PSED emphasizing the need for standardized neurological evaluations at follow‐up.

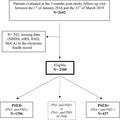

## 1 INTRODUCTION

Stroke is the main neurological cause of impaired quality‐adjusted life years (GBD 2016 Stroke Collaborators, 2019). While the severity of neurological symptoms and related disability account for a large proportion of the loss of functional independence and impaired quality of life of the patients and their relatives, additional poststroke complications significantly contribute to exacerbate its consequence (Broussy et al., 2019). Indeed, following stroke patients are prone to develop cognitive impairment, movement disorders, epileptic seizures, pain, sleep disorders, and emotional disturbances that are all potential sources of impaired functional outcome (Feyissa et al., 2019; Hackett et al., 2014; Harrison & Field, 2015; Lai et al., 2018; Mehanna & Jankovic, 2013; Mijajlović et al., 2017). To face this problem, the French health authorities have published in 2015 a decree indicating that every stroke patient should be evaluated 2 to 6 months after symptoms onset to assess the evolution of stroke symptoms and the occurrence of poststroke complications, in addition to address the questions of the stroke mechanism and efficacy and tolerance of stroke secondary prevention strategies (http://circulaire.legifrance.gouv.fr/pdf/2015/08/cir_39923.pdf).

Poststroke depression (PSD) and anxiety (PSA) represent the two main sources of poststroke emotional distress (PSED) (Robinson & Jorge, 2016; Towfighi et al., 2017). With an incidence varying between 20% and 65% of stroke survivors depending upon the population studied, stroke characteristics and the method of assessment used, PSED is an important source of imbalance between stroke‐related neurological disability and the severity of impaired quality of life. While this negative outcome is strongly related to the severity of PSD and PSA and might even be more significant among patients having comorbid mood and anxiety disorders, this issue is overlooked.

Several predictors of poststroke depression (PSD) and anxiety (PSA) have been consistently reported in the literature including younger age, female gender, a previous history of mood and/or anxiety disorders, a previous history of stroke, stroke‐induced severity of disability and aphasia, early depressive symptoms, and tobacco consumption (Almhdawi et al., 2020; Kutlubaev & Hackett, 2014; Perrain et al., 2020). The benefit of early selective serotonin reuptake inhibitor (SSRI) medication in the prevention of PSED has not been demonstrated unequivocally (Kim et al., 2017; Villa et al., 2018). Although an increased bleeding risk with SSRIs was suggested by retrospective studies, 3 large sample‐sized randomized studies performed among stroke patients (FOCUS, AFFINITY, and EFFECTS) did not confirm this suggestion. These studies have, however, emphasized two main points: first, the tolerance of SSRI is questionable, with a potential higher risk of bone fracture, hyponatremia, or epileptic seizure; and second, the use of SSRI seems to be associated with a lower incidence of depression. Altogether these data suggest that a better selection of stroke patients with a high risk of PSED and low risk of SSRI‐related complications is mandatory before starting these treatments. In addition, the benefit of pharmacological treatments in patients with identified PSED is variable across subjects, suggesting that these emotional symptoms might encompass several mechanisms. In this context, the association between poststroke complications and the presence of comorbid PSD and PSA (PSED) has been under‐investigated so far.

The aim of this study was thus to investigate in a stroke population evaluated at the 3‐month follow‐up visit the main determinants of stroke outcome associated with the presence of comorbid mood and anxiety disorders, a condition that we defined as poststroke emotional distress (PSED).

## 2 MATERIAL AND METHODS

### 2.1 Study design and ethics

This was a retrospective analysis of available electronic health records (EHR) from consecutive patients evaluated during a standard care poststroke follow‐up visit performed at Bordeaux University Hospital 3 to 4 months from the time of stroke hospitalization. The study population is part of the ObA2 regional cohort (National commission for data protection CNIL authorization n°911201) and each patient provided oral consent for the use of clinical, biological, and imaging data as collected in standard care. The study protocol and procedure for obtaining informed consent complied with the Helsinki declaration.

### 2.2 Sampling & study variables

This naturalistic study used a convenience sampling from stroke patients treated between the 1st of January 2016 and the 31st of March 2019. The main inclusion criteria were as follows: history of ischemic or hemorrhagic stroke confirmed on MRI or CT scan performed at symptoms onset (3 to 4 months before the follow‐up visit), age over 18 years old. Data were abstracted from Bordeaux University Hospital EHR system and included NIH stroke scale (NIHSS, [Brott et al., 1989]) modified Rankin scale (mRs, [van Swieten et al., 1988]), Hospital Anxiety‐Depression scale (HAD, [Zigmond & Snaith, 1983]), and Montreal Cognitive assessment (MoCA, [Nasreddine et al., 2005]) scale, demographic characteristics, vascular risk factors (hypertension, diabetes mellitus, obesity, alcohol, and tobacco consumption), history of previous stroke or TIA, history of heart disease (including ischemic heart disease, atrial fibrillation, and heart insufficiency), acute treatment (intravenous thrombolysis or endovascular therapy), and stroke mechanism according to the TOAST classification (Adams et al., 1993), and the presence or absence of pain, fatigue/fatigability, sleep problems, spasticity, abnormal movements, and de‐novo epilepsy at the follow‐up neurological evaluation.

### 2.3 Study population

Based on the findings from Bjelland et al. (Bjelland et al., 2002) as well as those from Burton & Tyson (Burton & Tyson, 2015) systematic review of mood screening tools for stroke survivors, the HAD was considered a satisfactory compromise in terms of clinical utility to screen for depression and anxiety in clinical routine in January 2016. The presence of PSD and PSA was defined, respectively, by an HAD‐Depression subscore and HAD‐Anxiety subscore greater than 7 (Zigmond & Snaith, 1983). Emotional status at follow‐up was dichotomized into a binary category (PSED+ vs. PSED−) based on the coexistence of these HAD defined PSD and PSA (PSED+) versus the absence of both (PSED−).

### 2.4 Statistical analyses

Quantitative variables were described using means, standard deviations (*SD*), and range values. Qualitative variables were described using counts and proportions.

Between‐group comparisons by emotional status at follow‐up (PSED+ vs. PSED−) were conducted with univariate analyses using Mann–Whitney and Chi2 for, respectively, quantitative (as their distribution was not normal) and qualitative variables.

The association between PSED and poststroke complications at 3 months was tested using a multivariate logistic regression model. The variables found to significantly distinguish the two groups in the univariate analyses were entered in the model; a *p* < .05 was considered statistically significant. All the statistical analyses were conducted using Jamovi 1.1.9.

## 3 RESULTS

**FIGURE 1:**
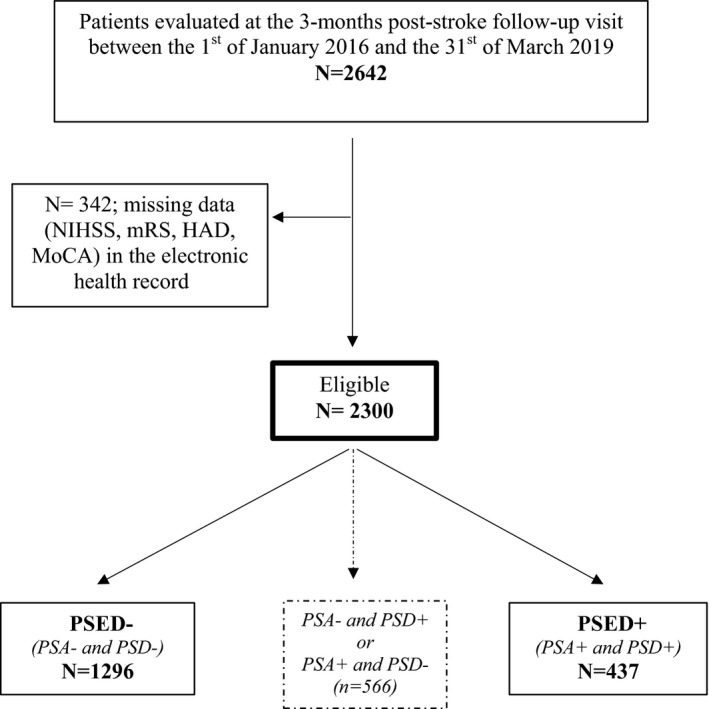
Study flowchart. PSA+ = Poststroke anxiety (HAD‐Anxiety ≥8); PSA− = No Poststroke anxiety (HAD‐Anxiety ≤7); PSD+ = Poststroke depression (HAD‐Depression ≥8); PSD− = No Poststroke depression (HAD‐Depression ≤7); PSED, Poststroke Emotional Distress.

The study flowchart is illustrated in Figure 1.

Out of the 2,642 records of patients evaluated at the 3‐month poststroke follow‐up visit during the study period, 2,300 (mean age 66.8 ± 15.1; 63.0% men) filled the predefined inclusion criteria and had no missing variables of interest.

Among these 2,300 patients, and according to the standard HAD cut‐off scores for depression and anxiety caseness, 28.0% had isolated PSD, 34.7% had isolated PSA, and 19% had comorbid depression and anxiety disorders (PSED+), leaving 56.39% of the population free of any these conditions (PSED−).

Only the PSED+ and PSED− participants were kept for the remaining analyses. Their main socio‐demographic and clinical characteristics are presented in Table 1. The majority of the overall sample was of male gender, with a level of education of less than 12 years, had a first‐ever stroke, a cerebral infarction and had hypertension. At the follow‐up visit, on average, the overall sample had a NIHSS of 1.23 ± 2.62, a mRs of 1.39 ± 1.28, a MoCA of 25.9 ± 4.21, and a HAD total score of 10.7 ± 8.40. Moreover, 36.2% had a persisting tobacco consumption and 13.1% had a persisting risky alcohol consumption. The most frequent poststroke complications were fatigue/fatigability (58.4%), sleep problems (26.7%), and pain (20.4%).

**TABLE 1:** Sample characteristics and univariate comparisons by poststroke emotional status

Demographic & Clinical characteristics	All (*n* = 1733)	PSED− (*n* = 1,297)	PSED+ (*n* = 437)	*p*‐value
Age, m ± *SD*	66.8 ± 14.9	67.13 ± 15.03	65.81 ± 14.65	0.028
Level of education <12y, *N* (%)	1,302 (75.1%)	981 (75.6%)	321 (73.5%)	0.362
Male, *N* (%)	1,124 (64.8%)	880 (67.8%)	244 (55.8%)	<0.001
First ever stroke, *N* (%)	1,473 (84.9%)	1,088 (83.9%)	385 (88.1%)	0.033
Previous TIA, *N* (%)	105 (6.1%)	68 (5.2%)	37 (8.5%)	0.015
Phasic disorder, *N* (%)	150^a^ (18.8%)	112 (18.4%) (*n* = 608)	38 (19.8%) (*n* = 192)	0.671
NIHSS at onset, m ± *SD* (range) (*n* = 1622)	3.43 ± 4.77	3.43 ± 4.72 (0–29)	3.45 ± 4.94 (0–32)	0.748
IVT, *N* (%)	277^b^ (25.4%)	195 (23.9%) (*n* = 816)	82 (29.9%) (*n* = 274)	0.047
EVT, *N* (%)	76 (4.4%)	59 (4.5%)	17 (3.9%)	0.561
**Stroke type, *N* (%)**				
Ischemic Stroke	1,275 (73.5%)	958 (73.9%)	317 (72.5%)	0.839
Hemorrhagic Stroke	329 (19.0%)	244 (18.8%)	85 (19.5%)	
TIA	130 (7.5%)	95 (7.3%)	35 (8.0%)	
**Vascular risk factors (admission), *N* (%)**				
Hypertension	985 (56.8%)	734 (56.6%)	251 (57.4%)	0.758
Dyslipidemia	665 (38.4%)	504 (38.9%)	161 (36.8%)	0.453
Diabetes	337 (19.4%)	252 (19.4%)	85 (19.5%)	0.992
Obesity	235 (13.6%)	167 (12.9%)	68 (15.6%)	0.156
Peripheral arterial disease, *N* (%)	74 (4.3%)	50 (3.9%)	24 (5.5%)	0.143
Heart disease, *N* (%)	404 (23.3%)	99 (22.7%)	305 (23.5%)	0.713
**3‐month poststroke evaluation**				
NIHSS, m ± *SD* (range)	1.23 ± 2.62	1.05 ± 2.53 (0–23)	1.79 ± 2.80 (0–17)	<0.001
MoCA, m ± *SD* (range)	25.9 ± 4.21	26.17 ± 4.02 (2–30)	24.92 ± 4.62 (3–30)	<0.001
mRS, m ± *SD* (range)	1.39 ± 1.28	1.19 ± 1.14 (0–5)	1.97 ± 1.48 (0–12)	<0.001
Fatigue, *N* (%)	1,013 (58.4%)	684 (52.7%)	329 (75.3%)	<0.001
Pain, *N* (%)	354 (20.4%)	216 (16.7%)	138 (31.6%)	<0.001
Sleep problems, *N* (%)	453 (26.7%)	277 (21.4%)	186 (42.6%)	<0.001
Epilepsy, *N* (%)	12 (0.7%)	7 (0.5%)	5 (1.1%)	0.187
Abnormal movements, *N* (%)	33 (1.9%)	17 (1.3%)	16 (3.7%)	0.002
**Consumption at follow‐up *N* (%)**				
Alcohol consumption	228 (13.1%)	168 (13.0%)	60 (13.7%)	0.678
Tobacco consumption	627 (36.2%)	447 (34.5%)	180 (41.2%)	0.011
Poly consumption	178 (25.7%)^c^	123 (24.6%) (*n* = 501)	55 (28.8%) (*n* = 191)	0.253
HAD total, m ± *SD* (range)	10.7 ± 8.40	6.41 ± 3.42 (0–14)	23.61 ± 4.89 (16–42)	<0.001
HAD Anxiety, m ± *SD* (range)	5.86 ± 4.29	3.76 ± 2.04 (0–7)	12.08 ± 2.98 (8–21)	<0.001
HAD Depression, m ± *SD* (range)	4.89 ± 4.50	2.65 ± 2.06 (0–7)	11.52 ± 2.96 (8–21)	<0.001
Antidepressant treatment, *N* (%)	474^d^ (29.1%)	358 (29.5%) (*n* = 1,215)	117 (28.1%) (*n* = 416)	0.604

Abbreviations: EVT, Endovascular Treatment; HAD, Hospital Anxiety and Depression Scale; IVT, Intravenous Thrombolysis; MoCA, Montreal Cognitive Assessment; mRS, modified Rankin Scale; NIHSS, National Institutes of Health Stroke Scale; TIA, Transient Ischemic Attack.

Heart disease = atrial fibrillation and/or heart failure and/or ischemic heart disease

Polyconsumption = more than one substance among tobacco, alcohol, and cannabis

^a^ *n* = 800

^b^ *n* = 1,090

^c^ *n* = 692

^d^ *n* = 1631

Compared to the PSED− group, PSED+ subjects were younger (mean difference of −1.36; Cohen's d = −0.09), more frequently female (*p* < .001; Cramer's V = 0.11), had more frequently a previous history of TIA (*p* = .015; Cramer's V = 0.06) but a first‐ever stroke (*p* = .033; Cramer's V = 0.05) and had more frequently a persisting tobacco consumption (*p* = .015; Cramer's V = 0.06), while no difference was observed in terms of other vascular risk factors and stroke mechanism. Neurological evaluation at follow‐up demonstrated that PSED+ patients had higher levels of symptom severity (NIHSS mean difference of 0.75; Cohen's d = 0.29), more severe functional disability (mRs mean difference of 0.78; Cohen's d = 0.63), lower cognitive abilities (MoCA mean difference of −1.25; Cohen's d = −0.30), and more frequently reported experiencing pain (*p* < .001; Cramer's V = 0.16), fatigue/fatigability (*p* < .001; Cramer's V = 0.20), sleep problems (*p* < .001; Cramer's V = 0.21), and abnormal movements (*p* < .002; Cramer's V = 0.07).

Multivariate analysis (Table 2) demonstrated that younger age (OR: 0.987), female gender (OR: 1.426), history of TIA (OR: 1.744), persisting tobacco consumption (OR: 1.360), higher functional disability (OR: 1.572), lower cognitive abilities (OR: 0.953), and experiencing sleep problems (OR: 2.334), pain (OR: 1.478), fatigue/fatigability (OR: 2.331), and abnormal movements (OR: 2.380) were all independently associated with the presence of PSED at the follow‐up visit. Conversely, NIHSS severity and a previous history of stroke did not remain significantly associated with the presence of PSED.

**TABLE 2:** Multivariate analysis of the 3‐month poststroke emotional status (PSED+ vs. PSED−)

Predictor	Estimate	SE	Z	*p*	Odds ratio	95% CI
Age	−0.13	0.00	−2.85	.004	0.987	0.98–1.00
Gender (women‐men)	0.35	0.13	2.82	.005	1.426	1.12–1.82
Previous TIA (Yes‐No)	0.56	0.23	2.37	.018	1.744	1.10–2.76
Previous stroke (Yes‐No)	−0.33	0.18	−1.81	.070	0.722	0.51–1.03
NIHSS	−0.03	0.03	−1.03	.302	0.973	0.92–1.02
mRs	0.45	0.07	6.75	<.001	1.572	1.38–1.79
MoCA	−0.05	0.01	−3.38	<.001	0.953	0.93–0.98
Tobacco consumption (Yes‐No)	0.27	0.13	2.02	.044	1.306	1.01–1.69
Sleep problems (Yes‐No)	0.85	0.13	6.60	<.001	2.334	1.82–3.00
Pain (Yes‐No)	0.39	0.14	2.74	.006	1.478	1.12–1.95
Fatigue (Yes‐No)	0.85	0.14	6.32	<.001	2.331	1.79–3.03
Abnormal movements (Yes‐No)	0.86	0.40	2.18	.029	2.380	1.09–5.17

Abbreviations: MoCA, Montreal Cognitive Assessment; mRS, modified Rankin Scale; NIHSS, National Institutes of Health Stroke Scale; TIA, Transient Ischemic Attack.

## 4 DISCUSSION

This study, which was performed in a large cohort of stroke patients evaluated in the setting of the daily clinical practice of a planned poststroke follow‐up visit, highlighted four main results: (a) the presence of comorbid poststroke depression and anxiety, a condition we termed poststroke emotional distress (PSED), was observed in about one in five patients, (b) stroke subtypes and mechanism were not associated with PSED, (c) most of the poststroke complications were independently associated with the presence of PSED, and (d) persisting tobacco consumption was the only vascular risk factor associated with PSED.

The prevalence of poststroke emotional distress we observed highlights that despite a growing literature on the risk to develop poststroke affective disorders, knowledge about early predictors, and data supporting the efficacy of antidepressant, a large number of patients still present PSED at follow‐up and this complication remains undertreated (Ladwig et al., 2018). These data should represent a call to health authorities and clinicians to optimize the follow‐up of stroke patients in the first weeks of stroke symptoms onset to improve the early detection and management of emotional disturbances. To this purpose, the development of new tools such as mobile applications already developed in the field of mental disorders to monitor patients will have to be explored (Jean et al., 2013; Johnson et al., 2009). These tools might even be more advisable to be used among patients with known early predictors such as young age, female gender, and more severe neurological deficit. Interestingly, our study also confirmed the strong association between a previous history of TIA and the presence of emotional disturbances at follow‐up (Kahlon & Nasrallah, 2019). Conversely, a trend for an opposite effect on PSED was observed for those who had a history of previous stroke. The interpretation of this result is questionable but it might be considered that those having a TIA will experience a fear of developing a new cerebrovascular event, potentially leading to a physical disability, lowering their threshold to develop affective disorders, while those with a history of stroke have already developed some resilience capabilities that reduce the emotional impact of a new stroke. Qualitative studies comparing the narratives of TIA and stroke patients could help to learn more on this issue.

Interestingly, we did not observe different rates of PSED depending on stroke mechanism, including cryptogenic stroke. This result suggests that most of the patients have less difficulties coping with the uncertainty about the cause of stroke compared to the strong impact of stroke‐related disability on daily‐life functioning. Yet, together with clinical data, several reports have described a potential role of biological and radiological biomarkers, such as stroke localization (Douven et al., 2017; Sutoko et al., 2020), on PSED. The role of these parameters on top of clinical data will thus deserve further analysis.

Although the early predictors of poststroke depression and anxiety have been previously investigated—including clinical, radiological, and biological factors (Nickel & Thomalla, 2017; Perrain et al., 2020; Wang et al., 2018)‐, few studies focused on the association between poststroke neurological complications and mood and anxiety disorders and even less as regards their comorbidity. Herein, we observed a strong association between the presence of PSED and the severity of physical disability and cognitive impairment but also the presence of sleep, pain, and movement problems. New tools have been developed to estimate the patient unmet needs, such as the Longer‐term Unmet Needs after Stroke questionnaire (LUNS, [Groeneveld et al., 2018]), which provide information that could help to inflect the patients' recovery trajectory. Here again, regular assessments of the patients' type and level of unmet needs using mobile technologies could help to tailor a personalized care program.

The association between functional dependency and PSED has been reported in several studies (Perrain et al., 2020; Towfighi et al., 2017). Interestingly, if baseline NIHSS scores did not differ between the two groups, NIHSS at follow‐up did, but we observed no significant independent association between the NIHSS measured at follow‐up and the presence of PSED in the multivariate analysis. This result strongly suggests that more than the symptoms severity itself, consequences on daily‐life activities are crucial factors and might indicate the difficulties of stroke patients to cope with these restrictions (Broussy et al., 2019).

The strong association between pain, whatever its location, and PSED is also in accordance with previous publications that demonstrated the negative impact of pain on stroke survivors' functional and mood outcome (Harrison & Field, 2015). Although the attention paid to pain by stroke patients might be increased by PSED, this finding highlights the need to incite the systematic evaluation of pain in stroke patients to provide specific management that might be more efficient than antidepressants alone on mood outcome.

As regards sleep disturbances and impaired cognition, all these manifestations are part of the depression or anxiety symptomatology so we cannot rule out that the significance of the association was a pure construct. However, we have to consider the bidirectional relationship between these variables and PSED. Indeed primary sleep disorders such as obstructive sleep apnea syndrome or vascular cognitive impairment are independent sources of impaired mood (Douven et al., 2018; Vanek et al., 2020). These strong associations still reinforce the need of a standardized evaluation of each potential source of PSED in order to optimize their pharmacological and nonpharmacological management.

In addition, the presence of movement disorders was also significantly associated with PSED. Unfortunately, this entity regrouped parkinsonism and movement disorders such as tremor or dystonia, which limited the interpretation of this finding. The severity of poststroke movement disorders is usually low and not supposed to be a direct source of PSED. Therefore, two main mechanisms can explain this association: the presence of apathy, which is often present in parkinsonism, and side effects related to antidepressant. These hypotheses will have to be more deeply investigated in further studies.

Together with neurological complications, we also observed that persisting tobacco consumption at stroke follow‐up visit was the only vascular risk factor associated with the presence of PSED. Over one third of the sample was concerned by this addictive behavior. This result is in line with the recent review from Perrain et al. that found an increased risk to develop poststroke depression among smokers (Perrain et al., 2020). Stroke patients are routinely asked about their consummatory behaviors in clinical practice and the frequency of current smokers, alcohol drinkers, and, more recently, cannabis users are regularly reported in the demographic characteristics of stroke cohorts. While the association between the intensity of substance use (quantities) and the risk of stroke has been demonstrated (Falkstedt et al., 2017; O'Donnell et al., 2016), the level of dependence to these substances is almost never reported though it might be a major limit in the quitting process that is systematically recommended in stroke secondary prevention. This observation suggests that patients who were not able to stop their consumption despite strong clinical advice and the use of nicotine replacement therapies (which usually correspond to those with a higher dependence to nicotine and related addiction) are more prone to develop PSED. This result is in line with the self‐medication hypothesis that conceptualizes addictions as “emotional anesthetics” (Khantzian, 1997) and strongly supports the need to conduct deeper evaluation of substance abuse in stroke patients to identify those with addictive behaviors that would require specific management.

Some results of the present study should be interpreted cautiously due to several limitations. First, although a large number of patients evaluated at the poststroke follow‐up visit were included in this study, those with severe aphasia or cognitive disorders who were unable to perform cognitive and emotional evaluations were excluded, which limits the generalizability of the results. Second, prestroke history of affective disorders and the emotional reaction in subacute stroke phase, which are two strong predictors of poststroke depression and/or anxiety (Sibon et al., 2012), were not recorded systematically. However, this study did not focus on the predictors of mood problems but on the association between poststroke neurological complications and psychological outcome therefore limiting the influence of these variables. Third, in addition to poststroke depression and fatigue, apathy is nowadays considered as an independent poststroke entity (Ferro et al., 2016) with specific consequences on stroke outcome. Because depression, fatigue, and apathy syndromes share some features, such as loss of energy or physical or mental slowing, we cannot exclude a misclassification of some patients. The role of apathy syndrome on stroke outcome on top of PSED and fatigue will require further investigations. Moreover, the classification of PSED was based on the results of the HAD score while the most appropriate approach for establishing a diagnosis of PSD or PSA should be a (semi‐)structured interview based on the clinical criteria listed in the standard classifications (DSM or ICD). Unfortunately, given our naturalistic setting, this evaluation was not possible due to limited human resources and our results strengthen the need to reinforce the healthcare system to optimize the management of stroke patients. Finally, we did not use standardized tools to quantify the dependence to substance use, therefore, limiting the interpretation of some results.

To conclude, this study confirms the high frequency of PSED at stroke follow‐up visit and illustrates the complex mechanisms of neuropsychological complications. A standardized evaluation of each poststroke complication and specific treatment combined to an evaluation of comorbid psychiatric disorders, such as substance use disorder, should become a standard of care to optimize poststroke neuropsychological management. This would allow a better identification of the determinants of PSED and an improvement of their therapeutic management, which will in turn reduce the societal burden of this complication.

## CONFLICT OF INTEREST

Igor Sibon has received fees for editorial activities with Elsevier, has served as advisor for Servier and Boehringer Ingelheim, has received teaching honoraria from Medtronic and Bayer, as well as research support from the University Hospital Bordeaux and the French Health Ministry. Sylvie Berthoz has received fees for editorial activities with Elsevier.

## AUTHOR CONTRIBUTIONS

Richard Houeze designed the study, acquired data, and wrote the first draft of the manuscript. Sagnier Sharmila, Olindo Stephane, Mathilde, Debruxelles Sabrina, and Rouanet François acquired data. Yolaine Rabat analyzed and interpreted the data, drafted the manuscript for intellectual content. Sylvie Berthoz and Igor Sibon designed and conceptualized the study, analyzed and interpreted the data, drafted and revised the manuscript for the intellectual content.

## INFORMED CONSENT AND PATIENT DETAILS

Patients provided oral consent and no personal details are included in any parts of the manuscript. All data were obtained in routine care in agreement with the Bordeaux University Hospital ethical review committee (approval ref GP‐CE‐ 2020–13). All the participants of the study were included in the “Observatoire Aquitain des AVC3” (ObA2 registry) supervised by the regional health agency of Nouvelle Aquitaine and having the requested authorization (National commission for data protection CNIL n°911201).

### PEER REVIEW

The peer review history for this article is available at https://publons.com/publon/10.1002/brb3.2158.

## Data Availability

The data and material that support the findings of this study can be accessed by reasonable request from the corresponding author.
